# Thermo-Acidic Pretreatment of Beach Macroalgae from Rügen to Optimize Biomethane Production—Double Benefit with Simultaneous Bioenergy Production and Improvement of Local Beach and Waste Management

**DOI:** 10.3390/md13095681

**Published:** 2015-09-03

**Authors:** Yann Nicolas Barbot, Laurenz Thomsen, Roland Benz

**Affiliations:** Department of Life Sciences and Chemistry, Jacobs University Bremen, Campus Ring 1, D-28759 Bremen, Germany; E-Mail: l.thomsen@jacobs-university.de; Department of Physics and Earth Sciences, Jacobs University Bremen, Campus Ring 1, D-28759 Bremen, Germany; E-Mail: r.benz@jacobs-university.de

**Keywords:** algae, biogas, beach management, bioremediation, eutrophication, pretreatment, waste treatment, Rügen, Baltic Sea

## Abstract

Eutrophication is a phenomenon which can rapidly generate masses of marine macroalgae, particularly in areas with high nutrient pollution. Washed ashore, this biomass impairs coastal tourism and negatively affects the coastal ecosystem. The present study evaluates the biochemical methane potential (BMP) of a macroalgae mix (Rügen-Mix, RM (RM = Rügen-Mix)) originating from Rügen, Germany. To improve biomethane recovery, thermo-acidic pretreatment was applied to the biomass prior to biomethanation to disintegrate the biomass macrostructure. Acid hydrolysis was successfully triggered with 0.2 M industry-grade HCl at 80 °C for a 2 h period, increasing biomethane recovery by +39%, with a maximum BMP of 121 mL·g^−1^ volatile solids (VS). To reduce the necessity for input material, HCl was replaced by the acidic waste product flue gas condensate (FGC). Improved performance was achieved by showing an increase in biomethane recovery of +24% and a maximum BMP of 108 mL·g^−1^ VS. Continuous anaerobic digestion trials of RM were conducted for three hydraulic retention times, showing the feasibility of monodigestion. The biomethane recovery was 60 mL and 65 mL·g^−1^ VS·d^−1^ for thermophilic and mesophilic operation, respectively. The quality of biomethanation performance aligned to the composition of the source material which exhibited a low carbon/nitrogen ratio and an increased concentration of sulfur compounds.

## 1. Introduction

### 1.1. Terrestrial Energy Crops for Biogas Production

Limited availability of fossil combustibles and the associated environmental, social, and political problems have led to the idea of providing energy from renewable sources to guarantee long-term energy security for domestic economies and societies [1,2,3]. Biogas is a renewable, biomass-based, gaseous energy carrier, similar to natural gas, and in the last decades, it has enjoyed intensive government promotion and private sector investments [4,5]. The many large-scale biogas plants found nowadays in Europe operate on energy crops such as maize silage (MS) and, hence, the quantitative requirements for biomaterials as substrate input have tremendously increased [6]. Intensified energy crop agriculture, necessary to serve the market demand, has led to a variety of environmental problems and social conflicts. These include the food-fuel debate [7], the excessive use of fertilizers and fresh water [8], soil leaching and soil erosion [9], threats to biodiversity sources [3,10,11] and conservation [9], additional greenhouse gas emissions [12], and the danger of monocultures [8]. Using marine or aqueous biomass, such as macroalgae, for biogas and biofuel production would circumvent many of these problems and allow a substantial detachment of food and biofuel production with regard to the raw biomaterial.

### 1.2. Anaerobic Digestion of Seaweeds

Compared with terrestrial plants, seaweeds show equal or superior qualities in serving as a source for biofuel production. Seaweeds lack lignin and contain only small quantities of cellulose [13], two compounds prevailing in the terrestrial biosphere which are extremely difficult to degrade by microorganisms [14]. Their chemical composition consists mainly of carbohydrates, many of which are easily fermentable, such as laminarin, mannitol, and starch [3,13]. Laboratory trials have shown good results for biomethane production from seaweeds [7,13,15]. Different mechanical, thermal, enzymatic, and thermo-chemical pretreatment methods have been shown to have a great effect on the improvement of the methane yield [16,17,18]. In particular, mechanical maceration and chopping [18,19] and thermo-chemical pretreatment at medium temperature (~80 °C) and moderate acidity range (0.15 M–0.2 M HCl) [20] are two seemingly effective techniques for biomass disintegration. Thermo-acidic pretreatment has been tested on macroalgal species, such as *L. japonica*, to increase the biomass saccharification and to improve the output of microbial hydrogen production, showing promising results [21,22,23]. Several other approaches that have been undertaken include co-digestion trials of macroalgae together with wheat straw or waste-activated sludge [16,24]. These tests aimed to improve anaerobic biomass digestion by combining C-rich (e.g., wheat straw) and N-rich (seaweeds) co-substrates to generate an optimized C/N ratio, suitable for the microorganisms involved in anaerobic digestion (AD) [25]. However, most of the studied macroalgal biomaterial consisted of a single macroalgae species [26,27] and biomethane recovery from native beached seaweed mixtures was rarely analyzed and only tested in batch experiments [28]. To our knowledge, extended experimentation in continuous laboratory- or pilot-scale applications, also with regards to long-term digestion, has not been thoroughly investigated.

### 1.3. Marine Biomass from Eutrophication-Afflicted Areas

Annual algal blooms are repeating events reported on the coastlines of Europe, the U.S., and Japan [19,29,30]. The accumulation of seaweed in the near-shore areas, the piling of seaweed on beaches, and its subsequent decomposition can present a threat to the marine ecosystem [28,31]. Furthermore, it impairs the touristic attractiveness of beach resorts due to foul smell and visual nuisance [32,33]. The Island of Rügen in Germany is located in the Baltic Sea and is a good example of a region, highly frequented by tourists, which suffers from regular eutrophication. Tourism, as an important sector of the local economy for the island, is a major source of income and resort operators are dependent on satisfied customers [34]. As a result, local authorities and hotel resorts must clear the seaweed biomass from the beaches, a process connected to high operating costs and resulting in the inappropriate dumping of biomass in deserted places or improper disposal [35,36]. This behavior might be connected to the lack of a defined legal framework for disposal of marine waste or the lax approach in implementing such a framework. A proper valorization of the recovered biomaterial, such as its use as a substrate in biogas plants, would greatly improve the economic concept of beach cleaning management [34]. The natural eutrophication process is greatly accelerated by human intervention when large quantities of inorganic effluents, agricultural run-offs, and maricultural waste are discharged into the sea [33,37]. This biomass could represent a potential substrate in biogas plants as has recently been suggested by many authors [19,33]. Different techniques to guarantee constant biomass supply and to monitor or predict the accumulation of seaweeds have been proposed, such as hydrodynamic models [38] and beach monitoring systems [30]. Barbot *et al.* (2014) [20] suggest a waste management concept in which beach macroalgal biomass is pretreated using other process waste products, such as flue gas condensate and waste heat from combustion processes, to increase the biomethane output. The accumulation of heavy metals in marine biomass has been detected in marine areas suffering from strong discharges of industrial wastes which lack proper distribution via tidal activity, such as the Baltic Sea shore [39]. Removal of macroalgae from strongly eutrophication- or hypertrophication-afflicted areas also provides the opportunity to reduce marine pollution through dissolved phosphate, nitrate, and heavy metals, thereby presenting an additional environmental benefit to this overall concept [33,40].

### 1.4. Aims of the Study

This study investigates the BMP and CH_4_ formation dynamics of the beached macroalgae blend Rügen-Mix to validate the potential biomethane recovery of this biomass. To increase the biomethane conversion efficiency, thermo-chemical acid hydrolysis pretreatment using industry-grade acid (HCl) and FGC is performed to enhance substrate degradation and to facilitate microbial conversion. As hydrolysis is known to represent a key step in the rate of the anaerobic digestion process, and macroalgae composition is susceptible to mild pretreatment conditions, this approach is expected to be sufficient for adequate presolubilization of RM biomass [20]. With the intention to supply current running biogas plants, frequently operating with maize silage, with macroalgal biomass, a co-digestion approach of RM together with maize silage is analyzed with respect to BMP and CH_4_ formation dynamics. Macroalgae are intended to partly replace a share of maize silage feedstock in order to reduce the quantity of maize silage required for biogas production. To determine the properties of long-term anaerobic digestion of RM and whether a difference in bioreactor operation temperature results in more promising biomethane conversion, the macroalgae are digested in mesophilic and thermophilic continuous mode for several different hydraulic retention times. Bearing in mind that the continuous anaerobic digestion process of RM will generate a constant flow of remaining digestate, the respective fermentation residue is analyzed with regard to its potential use as fertilizer. A valorization of the fermentation residue prevents the disposal of an additional waste product during the whole process.

## 2. Results

### 2.1. Composition of Rügen-Mix and Theoretical Methane Potential

An elementary analysis of Rügen-Mix to determine the concentration of macro- and micronutrients, as well as the quantity of heavy metals, was performed and values were matched with the limiting concentrations according to the German Biowaste Act *Bioabfallverordnung* (BioAbfV) (Table 1).

**Table 1:** Elementary analysis of RM biomass and literature values of elemental composition of maize.

Category	Element	Rügen-Mix	Maize [41,42]	Unit
Heavy metals (BioAbfV)	Lead (Pb)	8.6	2	mg·kg^−1^ TS
	Cadmium (Cd)	3.2	0.7	
	Chromium (Cr)	13	0.5	
	Copper (Cu)	20	4.5–5	
	Nickel (Ni)	15	5	
	Mercury (Hg)	0.05		
	Zinc (Zn)	141	35–56	
Macronutrients	Phosphorous (P)	1900	2200	
	Potassium (K)	11,400	17,800	
	Magnesium (Mg)	6030	2700	
	Calcium (Ca)	16,200	4500	
	Sulfur (S)	19,800	2700	
	C/N ratio	8.75:1	~30:1	
	Total carbon	35	43	% TS
	Total nitrogen	40,000	14,000	mg·kg^−1^ TS
Micronutrients	Molybdenum (Mo)	1.2	0.3	mg·kg^−1^ TS
	Iron (Fe)	6200	184	
	Cobalt (Co)	1.2	65	
	Selenium (Se)	0.8		
	Manganese (Mn)	180	29	

The BioAbfV represents a legal framework and deals with the recycling of biowaste in agriculture, forestry, and horticulture soils. Literature values for the composition of maize were taken to obtain comparable benchmark values [41,42]. For all measured concentrations of heavy metals, RM shows significantly higher values (three to 24 times) than maize, indicating a higher heavy metal contamination of marine biomass compared to terrestrial biomass. RM contains more magnesium, calcium, sulfur, and nitrogen, while maize shows higher concentrations of phosphorous, potassium, and carbon. The C/N ratios were 8.75:1 for RM and ~30:1 for maize, respectively. Regarding the levels of trace elements, RM contains greater amounts of iron, molybdenum, and manganese than maize, where, in particular, iron presents a much higher concentration (33 times higher). Only cobalt shows a higher concentration in terrestrial maize as compared to marine RM.

The composition of Rügen-Mix was analyzed for the shares of carbohydrate, protein, and lipids in the biomass in order to identify and characterize the substrate in use. RM is composed of 31.1% inorganic and 68.9% organic matter, of which 54% are carbohydrates, 24.4% proteins, and 0.2% lipids (Table 2). To obtain a maximum benchmark for the biomethane conversion output efficiency, the theoretical BMP from the total Rügen-Mix was calculated based on the BMP of each of the individual shares of carbohydrate, protein, and lipid as stated by the Bayrisches Landesamt für Umwelt (LfU) [43]. The LfU represents a state office for the environment and is under the jurisdiction of the Bavarian State Ministry for the Environment and Consumer Protection. The LfU collects and evaluates laboratory- and industry-scale data on biomethane recovery from different substrate types with respect to their composition. The predictive data presented by the LfU mirrors the effective biomethane yield obtained if optimal conversion is possible fairly well.

**Table 2:** Composition and theoretical methane potential of Rügen-Mix biomass.

Fraction	Component	Share [%]	Theoretical CH_4_	Unit
Volatile solids		68.9		
	Carbohydrate	44.3	164	mL·g^−1^ TS
			238	mL·g^−1^ VS
	Fiber	9.7		
	Protein	24.4	112	mL·g^−1^ TS
			163	mL·g^−1^ VS
	Lipid	0.2	2	mL·g^−1^ TS
			3	mL·g^−1^ VS
Inorganic solids		31.1		
Total		100	278	mL·g^−1^ TS
			404	mL·g^−1^ VS

A fixed rate of 7% was subtracted from the theoretical value to account for biomass generation through microbial growth during the experiment not being available for biomethane formation [44]. Combining results from RM composition analysis, LfU data, and VDI 4630 (Technical guideline rule, “Characterisation of the substrate, sampling, collection of material data, fermentation tests” by the Verein Deutscher Ingenieure), the total theoretical biomethane recovery from RM sums up to 278 mL·g^−1^ TS, or 404 mL·g^−1^ VS (Table 2).

### 2.2. BMP of Acid Hydrolysis Pretreated Rügen-Mix

**Figure 1:**
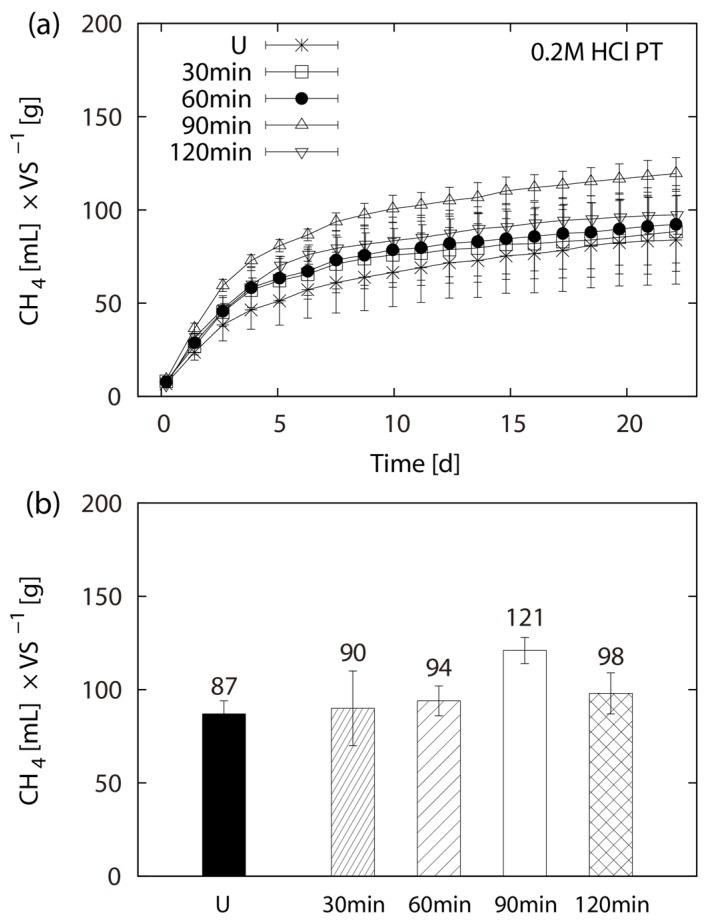
Reaction time of thermo-acidic pretreatment: Net accumulated methane production of pretreated and untreated (U) Rügen-Mix. Pretreatment was applied for 30 min, 60 min, 90 min, and 120 min at 80 °C in 0.2 M HCl (**a**). (**b**) shows the histogram of the final net methane yield in mL·g^−1^ VS algae biomass. The values of the final methane yield are reported in Table 3 and were taken at Day 22.

The total biomethane yield of non-pretreated RM resulted in 87 mL·g^−1^ VS. Pretreatment of RM with 0.2 M HCl at 80 °C for 30 min, 60 min, 90 min, and 120 min yielded 90 mL, 94 mL, 121 mL, and 98 mL CH_4_·g^−1^ VS, respectively (Figure 1a,b). The pretreatment reaction time of 90 min showed the best performance, increasing the BMP by +39% when compared to untreated biomass. Changing the acid concentration of the pretreatment medium showed that a concentration of at least 0.1 M is necessary to achieve improvement in BMP.

**Figure 2:**
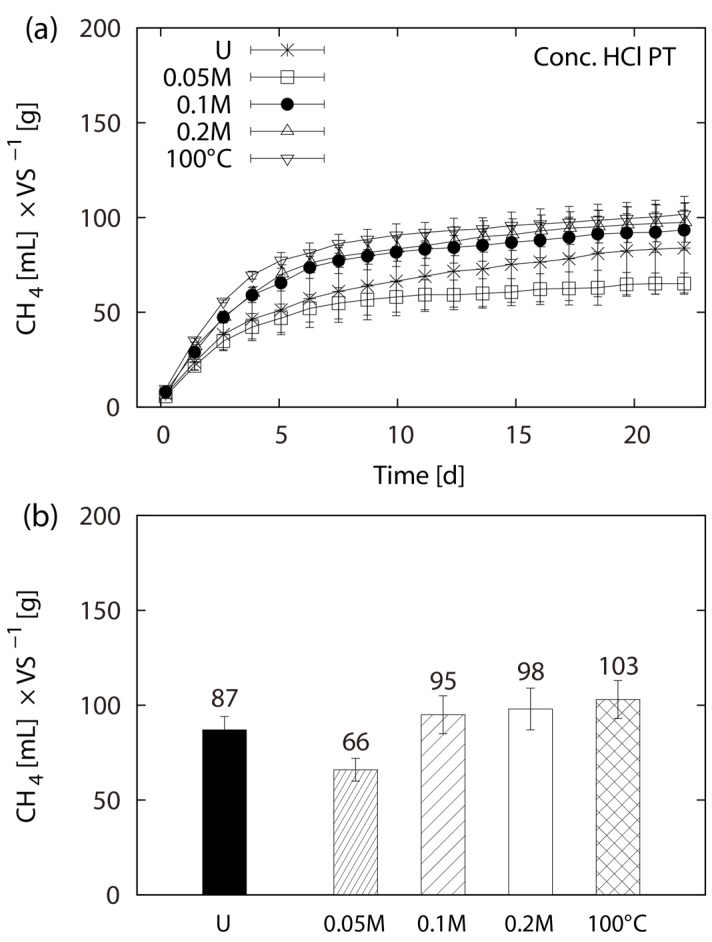
Acid concentration for pretreatment: Net accumulated methane production of pretreated and untreated (U) Rügen-Mix (**a**). Pretreatment was applied at 80 °C for 2 h in 0.05 M (0.05 M), 0.1 M (0.1 M), and 0.2 M HCl (0.2 M) and at 100 °C for 2 h in 0.2 M HCl (100 °C); (**b**) shows the final net methane yield in mL·g^−1^ VS algae biomass. The values of the final methane yield are reported in Table 3 and were taken at Day 22.

**Figure 3:**
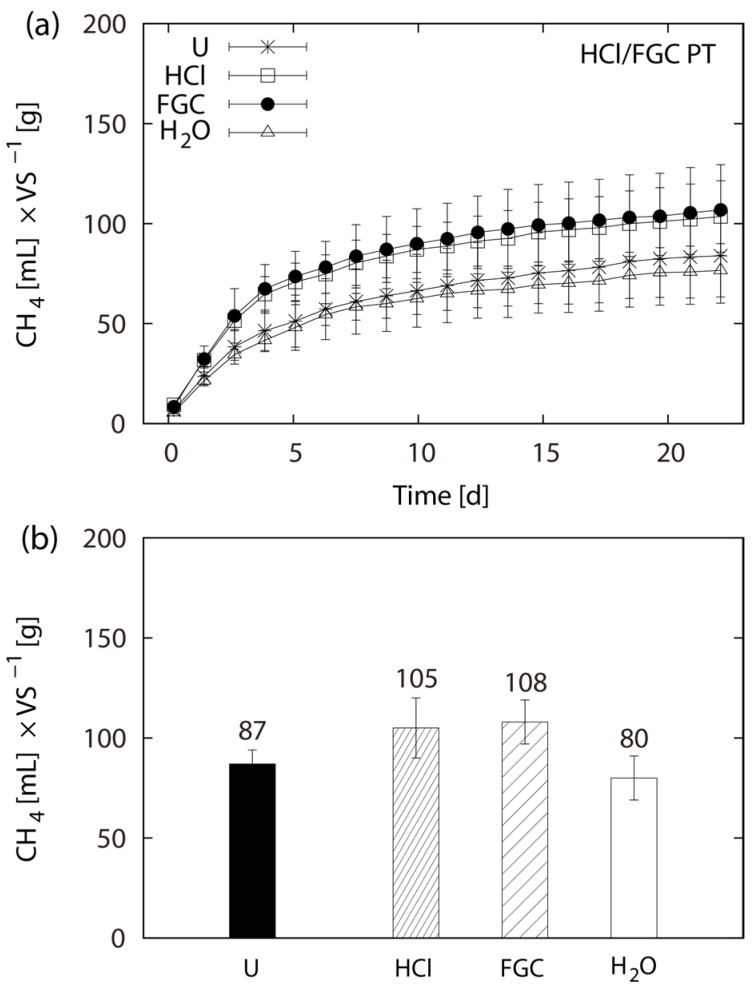
Thermo-acidic pretreatment with FGC: Net accumulated methane production of pretreated and untreated (U) Rügen-Mix. Pretreatment was applied for 2 h at 80 °C in HCl, pH 1.2 (HCl), and FGC, pH 1.2 (FGC), and water (H_2_O) (**a**). (**b**) shows the histogram of the final net methane yield in mL·g^−1^ VS algae biomass. The values of the final methane yield are reported in Table 3 and were taken at Day 22.

Pretreatment with 0.05 M, 0.1 M, and 0.2 M HCl at 80 °C resulted in biomethane yields of 66 mL, 95 mL, and 98 mL·g^−1^ VS, respectively (Figure 2a,b). Increasing the pretreatment reaction temperature from 80 °C to 100 °C in 0.2 M HCl showed a BMP of 103 mL·g^−1^ VS. The improvement effect on biomethane production with 0.05 M to 0.2 M HCl, with simultaneous heat application (80 °C), is maximum +13%. RM pretreated with H_2_O, FGC (pH 1.2), or HCl (with pH adjusted to that of FGC = pH 1.2) showed BMPs of 80 mL, 108 mL, and 105 mL·g^−1^ VS, respectively (Figure 3a,b). Hydrothermal pretreatment showed even poorer results in BMP than untreated biomass. Using FGC instead of HCl media for pretreatment achieved a similar quality of pretreatment performance with regards to total BMP, with an improvement of +24% compared to untreated RM. Comparing real BMPs with the theoretical methane yield calculated from the RM, the degradation efficiency was 22% for untreated RM, 27% for pretreated RM in FGC swb (at 80 °C for 2 h), and up to 30% for pretreated RM in 0.2 M HCl (at 80 °C for 90 min).

**Table 3:** Kinetic decay constants (K) and related values of degradation dynamics (time until 50%, 70%, and 90% of maximum BMP (T_50_, T_70_, T_90_) were produced) for all batch experiments. Name of the sample (Name), pretreatment medium and concentration (Medium), pretreatment conditions (PT conditions), and maximum BMP are stated with standard deviation (SD).

Name	Medium	PT conditions	K (d^−1^)	T_50_	T_70_	T_90_	BMP [mL·g^−1^ VS]	SD
U	RM untreated	-	0.2145	3.7	6.4	12.3	87	±7
RM-U	RM untreated	-	0.1912	3.6	6.3	12.0	96	±4
0.05 M	0.05 M HCl	80 °C/2 h	0.2899	2.4	4.2	7.9	66	±6
0.1 M	0.1 M HCl	80 °C/2 h	0.2591	2.7	4.6	8.9	95	±10
0.2 M	0.2 M HCl	80 °C/2 h	0.2476	2.8	4.9	9.3	98	±11
100 °C	0.2 M HCl	100 °C/2 h	0.305	2.8	4.9	9.4	103	±10
30 min	0.2 M HCl	80 °C /30 min	0.2662	2.6	4.5	8.6	90	±20
60 min	0.2 M HCl	80 °C/60 min	0.2536	2.7	4.7	9.1	94	±8
90 min	0.2 M HCl	80 °C/90 min	0.2442	2.8	4.9	9.4	121	±7
HCl	HCl pH 1.2	80 °C/2 h	0.2467	2.8	4.9	9.3	103	±10
FGC	FGC pH 1.2	80 °C/2 h	0.2477	2.8	4.9	9.3	108	±11
H_2_O	H_2_O	80 °C/2 h	0.1976	3.5	6.1	11.7	80	±11
MS-U	MS untreated	-	0.2543	2.7	4.7	9.1	303	±37
50/50	MS/RM 50%/50%	-	0.2428	2.9	5.0	9.5	210	±16
75/25	MS/RM 75%/25%	-	0.2628	2.6	4.6	8.8	255	±31

### 2.3. BMP of Rügen-Mix Co-Digested with Maize Silage

**Figure 4:**
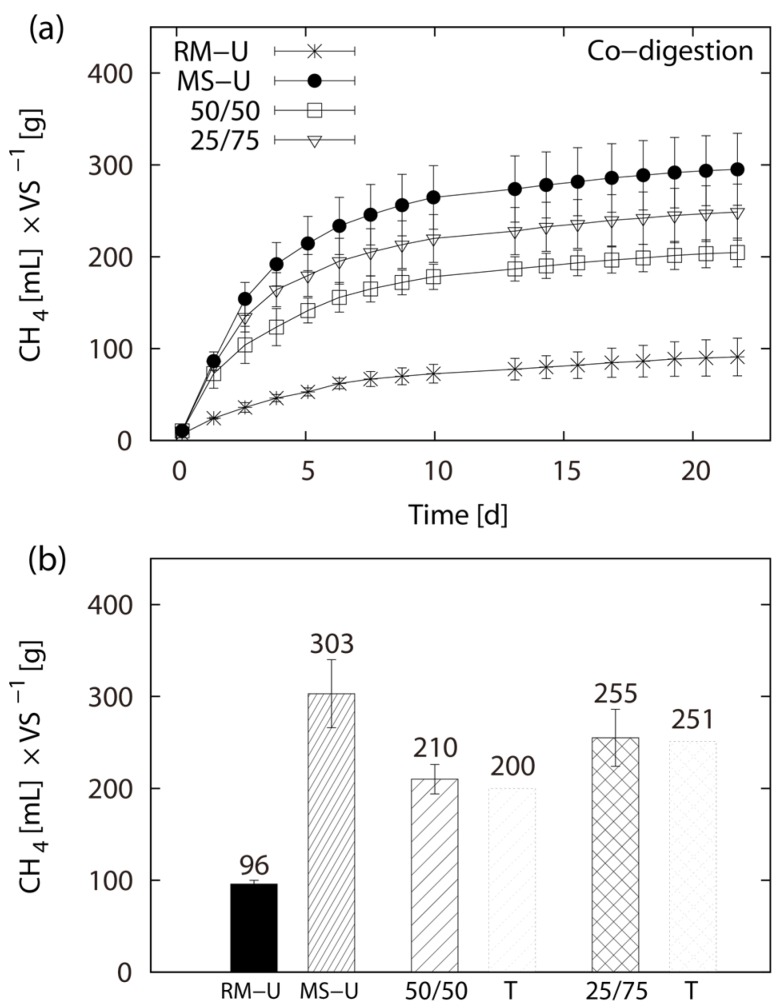
Co-digestion with maize silage: Net accumulated methane production of monodigestion from Rügen-Mix (RM-U), maize silage (MS-U), and their respective blends, 50% RM/50% MS (50/50) and 25% RM/75% MS (25/75) (**a**). (**b**) shows the histogram of the real final net methane yield in mL·g^−1^ VS algae biomass, along with the theoretical BMPs (T). The values of the final methane yield are reported in Table 3 and were taken at Day 22.

Single anaerobic digestion of MS and RM yielded 96 mL and 303 mL CH_4_·g^−1^ VS, respectively, while the 50/50 and the 75/25 blends of MS/RM showed biomethane recoveries of 210 mL and 255 mL·g^−1^ VS, respectively (Figure 4a,b). The theoretical biomethane yields of the blends, which were based on calculations of the results from single digestion, sum up to 200 mL and 251 mL·g^−1^ VS. Thus, the real data indicates a slight increase in biomethane recovery (respectively 5% and 2%) when compared to the theoretical calculations.

### 2.4. Evaluation of Methane Production Dynamics

The rate of methane formation was determined for the respective batch experiments using the formulas stated in Section 4.7.3. Therefore, three reference milestones were set in the batch process, displaying the time needed until 50%, 70%, and 90% of the maximum BMP (taken after Day 22) was produced. These reference points for the different batch experiments (Table 3) were compared to each other and the findings are integrated in the respective sections of the discussion.

### 2.5. Continuous Reactor Studies: Mesophilic AD

**Figure 5:**
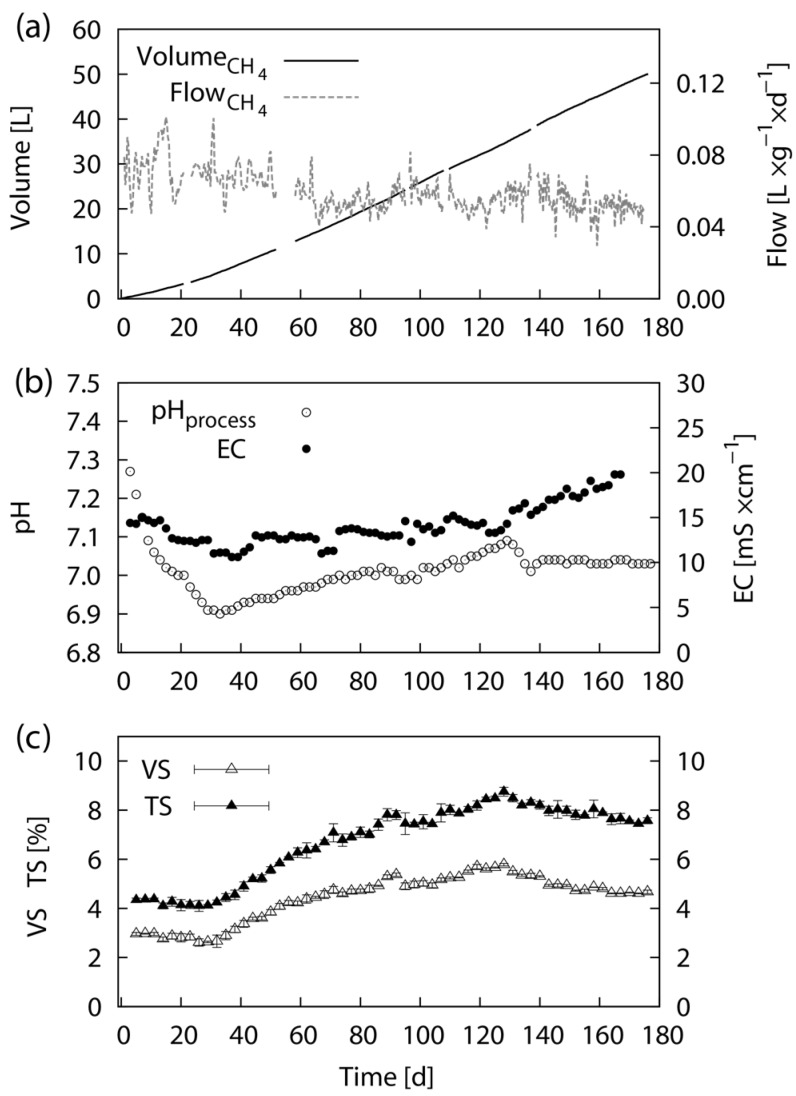
Continuous fermentation at mesophilic operation: Mesophilic continuous AD of untreated Rügen-Mix showing the process parameters; cumulative CH_4_ volume [L], specific volumetric CH_4_ production (calculated) per VS feed load (L·g^−1^·d^−1^) (**a**), pH value and conductivity EC in mS·cm^−1^ (**b**), VS and TS concentrations (% from total weight) (**c**). VS and TS represent average values from three individual measurements and error bars are defined by ±SD.

The specific CH_4_ flow rate showed a steady and slow decline from 80 mL to 55 mL·g^−1^ VS·d^−1^ over the 178 days of the experiment, with an average of around 60 mL·g^−1^ VS·d^−1^ (Figure 5). Overall, around 50 L CH_4_ were produced and a total of 764 g VS (1109 g TS) of RM biomass was used, producing an average CH_4_ yield of 65 mL·g^−1^ VS (45 mL·g^−1^ TS). The respective average CH_4_ yields for the different phases of bioreactor operation were 73 mL, 68 mL, 62 mL, 54 mL, and 53 mL·g^−1^ VS·d^−1^ (Table 4). The pH in the bioreactor showed a continuous drop from an initial pH of 7.3 to pH 6.9 at Day 32. Then, a recovery was observed where the values slowly increased to pH 7.09 at Day 128 and leveled out thereafter around pH 7.0. The conductivity slowly increased from an initial value of 15 mS·cm^−1^ to 20 mS·cm^−1^ at the end of the experiment. The VS and TS concentrations began to increase in accordance with increasing organic loading rate (OLR), from 2.8% to 5.8% (VS) and from 4% to 8.7% (TS). Subsequently, with the change in hydraulic retention time (HRT) (Day 131), they slowly decreased and leveled out at 4.5% (VS) and 7.5% (TS).

**Table 4:** Mesophilic and thermophilic operating conditions of the bioreactors with their respective specific CH_4_ production rates.

Mode	Phase	Time [days]	CH_4_ production [mL·g^−1^·d^−1^ VS]	OLR [g·L^−1^·d^−1^]	HRT [d]
Meso	P1	4–21	73	1.0	40
	P2	22–35	68	1.5	40
	P3	36–69	62	2.0	40
	P4	70–132	54	2.5	40
	P5	133–175	53	2.5	31
Thermo		4–57	65	3.0	15

### 2.6. Continuous Reactor Studies: Thermophilic AD

**Figure 6:**
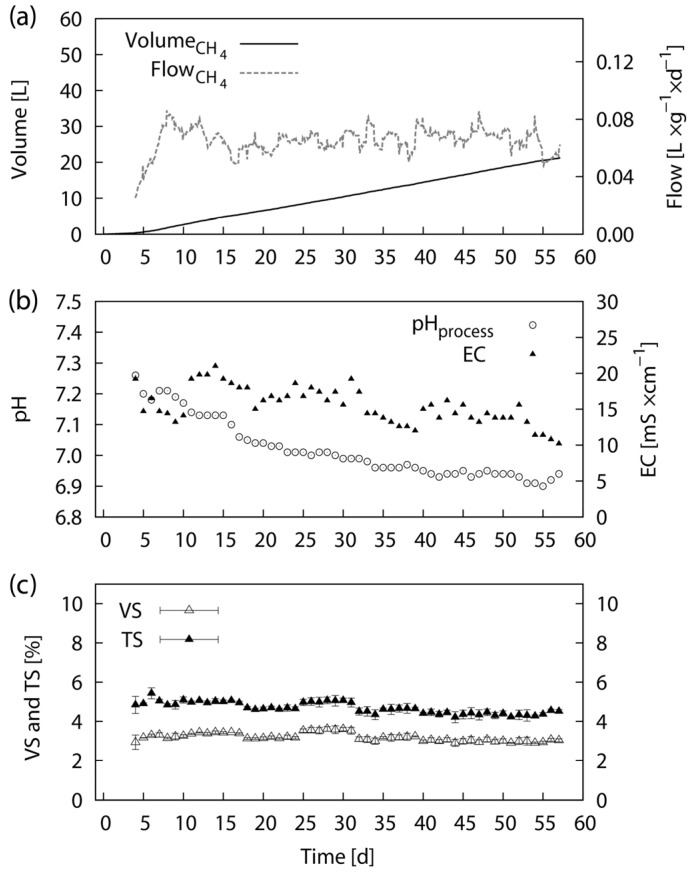
Continuous fermentation at thermophilic operation: Thermophilic continuous AD of untreated Rügen-Mix showing the process parameters; cumulative CH_4_ volume [L], specific volumetric CH_4_ production (calculated) per VS feed load (L·g^−1^·d^−1^) (**a**), pH value and conductivity EC in mS·cm^−1^ (**b**), VS and TS concentration (% from total weight) (**c**). VS and TS represent average values from three individual measurements and error bars are defined by ±SD.

Over the 58 days of the experiment, around 21.3 L CH_4_ were produced and a total of 324 g VS (470 g TS) of RM biomass was used as feedstock, providing an average CH_4_ yield of 66 mL·g^−1^ VS (45 mL·g^−1^ TS) (Figure 6). HRT was 15 days and OLR was 3.0 g VS·L^−1^·d^−1^ throughout the entire experiment (Table 4). The values for pH decreased steadily from an initial pH of 7.28 to pH 6.9, until Day 55. Subsequently, the values ceased to decline and again showed a small increase, remaining around pH 7.04 until the end. The conductivity slowly decreased from 20 mS·cm^−1^ to 10 mS·cm^−1^ over time. The VS and TS concentrations were almost steady throughout the entire experiment at ~3.2% (VS) and ~4.7% (TS).

### 2.7. Analysis of Fermentation Residue

An elementary analysis of RM fermentation residue was conducted to check its compositional properties and the results were compared to the values of the RM substrate, the energy crop digestate (as a reference benchmark), and the declaration limit for fertilizer application according to the German Düngemittelverordnung (DüMV) [45] (Table 5). The DüMV is part of the German Fertilizer Ordinance and presents a legal framework which regulates the authorization and the labeling of the fertilizer used. The aim of the Fertilizer Ordinance is a sparing use of fertilizer, a reduction of nutrient losses, and a reduction of nutrient discharges into rivers and other ecosystems. The concentration of macronutrients in RM and RM fermentation residue (RMR) were similar except for nitrogen and carbon, which showed reduced (−50%) and elevated (42% from TS) quantities in RMR, respectively. The C/N ratio as well as total carbon, nitrogen, and calcium concentrations in RMR were comparable to that of the energy crop digestate [45]. As anticipated from the high sulfur concentration in the substrate RM, the sulfur quantity in RMR was four times higher than in the reference digestate. Among micronutrients, only iron showed a much higher concentration in RMR than in energy crop digestate and was approximately 85-fold higher than the declaration limit according to the DüMV.

The concentrations of heavy metals were generally above those of common digestate, but below the legal limitation values. This was true except for cadmium, in which the concentration exceeded the limiting concentration for fertilizer application by two-fold.

**Table 5:** Macro- and micronutrient composition and heavy metal concentration of RM, RMR, average energy crop digestate (digestate) [45], and the limiting concentrations for component declaration according to DüMV.

Category	Element	RM	RMR	Digestate	DüMV	Unit
Heavy metals (BioAbfV)	Lead (Pb)	8.6	11	2.9	150	mg·kg^−1^ TS
	Cadmium (Cd)	3.2	3.8	0.26	1.5	
	Chromium (Cr)	13	21	9.0	300	
	Copper (Cu)	20	28	69	70	
	Nickel (Ni)	15	20	7.5	80	
	Mercury (Hg)	0.05	0.04	0.03	1	
	Zinc (Zn)	141	215	316	500	
Macronutrients	Phosphorous (P)	1900	2800	25,700	300	
	Potassium (K)	11,400	14,800	71,400	500	
	Magnesium (Mg)	6030	8120	12,000	300	
	Calcium (Ca)	16,200	21,300	30,000	500	
	Sulfur (S)	19,800	21,300	4710	300	
	C/N ratio	8.75:1	10:1	6.4:1		
	Total carbon	35	42	43		% TS
	Total nitrogen	40,000	21,300	67,140	1000	mg·kg^−1^ TS
Micronutrients	Molybdenum (Mo)	1.2	1.6		2	mg·kg^−1^ TS
	Iron (Fe)	6200	8560		100	
	Cobalt (Co)	1.2	2.9		4	
	Selenium (Se)	0.8	1			
	Manganese (Mn)	180	280		200	

## 3. Discussion

### 3.1. Biomass Composition and Theoretical BMP

The Rügen-Mix biomass was mainly composed of eelgrass (*Zostera marina*) and green (*Chaetophorales* family) and red macroalgae (*Spermothamnion* family), with the latter providing the largest share. The composition of red algae is very diverse across the species but they generally contain agar, carrageenan, glucan, galactan, and cellulose and mostly feature high carbohydrate content [13]. The sugar composition consists mainly of galactose (hydrolysis product of agar) and glucose sub-units [7]. Green algae are composed of starch, cellulose, and pectin [13]. Carrageenans and pectin exhibit gel-forming ability [7,13] and this effect was partially observed in the consistency of bioreactor sludge during the experiments. Considering the usually pre-dominant content of carbohydrates in macroalgae, this biomass also contained a high quantity of proteins. The degradation efficiency of RM ranged between 22%–30% compared to its theoretical counterparts. In all cases, a considerable part of the organic matter of RM was not converted to methane. The diminished conversion efficiency might have been related to the biomass composition. Larger concentrations of sulfur compounds were found in the substrate and digestate which align with the findings of Migliore *et al.* (2012) [28] for beached macroalgae from Orbetello Bay, Italy. The high sulfur concentration possibly originates from carrageenan, consisting of alternating sulfated d-galactose subunits [13], or sulfated polysaccharides in the form of hetero-hexanes and pentanes [28]. Subsequently, during decomposition, microbial inhibition takes place due to cell toxicity from the increased presence of H_2_S [46]. Also, the C/N ratio of RM was 8.75:1, hence considerably lower than the optimal range for AD, which might have intensified the microbial inhibition effect [25]. The quantities of macronutrients were comparable to those of maize, with superior concentrations for nitrogen, magnesium, and calcium. Magnesium and calcium, in particular, might positively act on AD. RM turned out to be rich in iron and molybdenum, with an almost 34-fold and four-fold greater quantity, respectively, compared to maize. A co-digestion scheme, together with C-rich and trace element deficient biomass, could hence complementarily improve the degradation quality of both co-substrates on a continuous basis. Molybdenum is usually the limiting element in AD of maize silage, while iron is the limiting element in AD of *Napiergrass* [47].

### 3.2. Effect of Acid Hydrolysis on BMP

Untreated RM biomass yielded 87 mL CH_4_·g^−1^ VS. Only few data on AD of beached macroalgae blends with comparable conditions and composition to RM were found in the literature. Migliore *et al.* [28] presented the AD of a macroalgae mix from the Lagoon of Orbetello consisting of green algae, *Chaetomorpha linum*, and red algae, *Gracilariopsis longissima*, yielding 380 mL CH_4_·g^−1^ VS. Other literature examples presented the CH_4_ yields for AD of single green and red macroalgae species involved in beach accumulation or eutrophication events. Allen *et al.* (2013) [19] yielded 226–250 mL CH_4_·g^−1^ VS when digesting dried, washed, and macerated *Ulva lactuca* and Oliveira *et al.* (2014) [48] obtained a BMP of 162–271 mL·g^−1^ VS and 481 mL·g^−1^ VS from methanation of fresh, macerated *Ulva lactuca* and washed, macerated *Gracilaria vermiculophylla*, respectively. In addition, Costa *et al.* (2012) [26] demonstrated a 196 mL·g^−1^ VS BMP for *Ulva* sp. When comparing these yields with untreated RM in this study, they appear considerably higher. The application of (acid hydrolysis) pretreatment to the biomass using industry-grade HCl or FGC, preceding AD, successfully increased the BMP as suggested by the hypothesis. Hydrothermal pretreatment (tap water) yielded a similar BMP as untreated RM, while acid hydrolysis pretreatment with FGC or HCl yielded up to +24% and +39% of CH_4,_ respectively, when compared to untreated biomass. The most beneficial process conditions for the improvement of biomethanation of RM were 0.2 M HCl, a pretreatment reaction temperature of 80 °C, and a pretreatment reaction time between 90–120 min. Lower temperatures or shorter reaction times resulted in diminished performance, while higher temperatures or longer reaction times did not improve the obtained output. The industry-grade HCl used for PT could perfectly be replaced by FGC swb (pH 1.2) while achieving almost similar performance in improving biomethanation. The effective conditions for PT using FGC swb (acidity, low pH after mixing biomass and media) were equitable to those of HCl. Only few articles in the literature deal with acid hydrolysis pretreatment of green and red macroalgae. Most involve applying acid hydrolysis or hydrothermal PT with the aim of improving macroalgal saccharification for bioethanol production. Dilute acid pretreatment of red algae, *Gelidiella acerosa*, induced a significant reduction of non-cellulosic solid compounds while simultaneously increasing the saccharification rate [49]. Hydrothermal treatment (HTT) of *Chaetomorpha linum* biomass at 180–200 °C caused high extractions of xylan and arabinan into the hydrolysate [50]. Although dealing with bioethanol production, the basic mechanism for both biomethanation and bioethanol production of carbohydrate-rich macroalgae is dependent on the availability of soluble sugars/nutrients. A part of the knowledge gained from the improvement of bioethanol production from macroalgae can be applied to the pretreatment process for biomethanation. However, in spite of the successful improvement of CH_4_ yield with the application of PT on RM, the total BMP was still situated in the bottom range compared to the results stated in the literature. The biomass composition analysis (high S concentration) and the shape of the CH_4_ production curves (delayed production pattern) suggest the presence of microbial inhibitor substances in the bioreactor responsible for average conversion efficiency.

### 3.3. Effect of Co-Digestion of Rügen-Mix with Maize Silage on Total BMP

Co-digestion of RM with maize silage showed an improved CH_4_ yield of +2% (25%/75%) and +5% (50%/50%), depending on the share of RM/MS blended. The co-digestion effect might be more prominent in continuous AD and long-term use regarding the benefits mentioned in the paragraphs above. In contrast to the findings in this work, the co-digestion of *U. lactuca* with dairy slurry (+17%) [51], *Gracilaria vermiculophylla* with glycerol [48], or *Ulva* sp. with waste activated sludge (+26%) [26] led to a more persuasive improvement of total BMP.

### 3.4. Effect of Acid Hydrolysis and Co-Digestion on Methane Formation Dynamics

Following acid hydrolysis, an acceleration of methane formation was noted, thus reducing the time required, by around three days, to reach the 90%-mark for total BMP (reached at Day 12 after feeding for untreated RM). Higher values for the kinetic decay constant indicate faster degradation rates (analog to faster biomethane formation rate). The improvement effect was similar in the acid hydrolysis pretreatment approaches, including with FGC swb. Hydrothermal PT only showed a minor improvement effect on formation dynamics, while increasing the PT reaction temperature from 80 °C to 100 °C in HCl additionally lowered CH_4_ formation time by 1–2 days. All decay constants of pretreated samples were higher than the untreated ones, with the highest value for pretreatment of RM in 0.2 M HCl at 100 °C for 2 h. These findings match the concept of enhanced saccharification with acid hydrolysis PT where readily fermentable sugars are solubilized from the solid biomass and dissolve in the hydrolysate, as suggested in this work and described by Montgomery and Bochmann (2014) [52]. The co-digestion of RM and MS did not significantly change the CH_4_ formation dynamics but rather showed an average trend of both single formation patterns. The methane formation for all approaches started almost instantly after feeding without showing a distinct substrate adjustment (Δ lag) phase. This suggests that a certain amount of readily fermentable compounds were available in the biomass from the beginning of AD.

### 3.5. Biomethane Production from Rügen-Mix during Mesophilic and Thermophilic Continuous Anaerobic Digestion

The long-time use of untreated RM in AD proved that monofermentation of RM was possible under mesophilic and thermophilic process conditions covering 2–3 hydraulic retention times. The average specific CH_4_ yields obtained were 60 mL·g^−1^ VS and 65 mL·g^−1^ VS for the mesophilic and thermophilic approach, respectively, showing that total CH_4_ recovery was only slightly higher in the thermophilic process operation.

Mesophilic operation showed differences in CH_4_ production profiles across the different stages towards the steady state conditions, with considerably changing daily biomethane recovery over time. The daily recovery was ~70 mL·g^−1^ VS within the first hydraulic retention time and 54 mL·g^−1^ VS after three hydraulic retention times. This decline in the CH_4_ production rate with ongoing experiment duration was also observed and described by Hinks *et al.* (2013) [53] for the AD of brown seaweed *Laminaria hyperborea*. The authors suggested the effect to be caused by a change of microbial community over time. The effect observed in this work might (additionally) originate from the accumulation of opportunistic inhibitory compounds in the bioreactor, such as sulfurous compounds, thoroughly present in the source biomass (see paragraph on biomass composition and digestate analysis). Current comparable data on the continuous monofermentation of red or green macroalgae is scarce in the literature. Prior works from Habig and Ryther (1984) [32] and Briand and Morand (1996) [54] showed CH_4_ production rates of 190 mL·g^−1^ VS for AD of *Gracilaria* sp. and 200–230 mL·g^−1^ VS for AD of *Ulva* sp. However, although exhibiting better performance, their bioreactors were operated at organic loading rate below 2.0 g and 1.0 g VS·L^−1^·d^−1^, which is considerably lower than in this study. The degree of degradation in mesophilic AD of RM was 41% and, thus, better than the VS reduction rate of 32% from Baltic Sea red algae presented by Biswas (2009) [55], but lower than the conversion rate of 38%–58% for *Ulva* sp. [32] and 73%–84% for maize silage [56]. The degree of degradation for thermophilic AD of RM was only 30% and was therefore even lower than for mesophilic operation. Allen *et al.* (2014) [51] tested the continuous digestion of *U. lactuca*, together with dairy slurry in different blending ratios and at moderate OLR. The authors discovered that high shares of macroalgae in the feed (50% VS) led to a disturbance in digester stability, a notable increase in total volatile fatty acid (VFA) concentration, and an increase in ammonia concentration. They suggested that high sulfur concentration might be responsible for these results. These observations are supported by the findings in this work where a high S concentration most likely led to diminished conversion performance, particularly for thermophilic AD, as the effect is reinforced by elevated temperature. The low C/N ratio of RM might also have played a role in diminished degradation. Allen *et al.* (2013) [19] observed that methanation was inhibited through low C/N ratio due to increased levels of ammonia and Habig *et al.* (1983) [54] demonstrated that the methane conversion rate of *Gracilaria tikvahiae* and *Ulva* sp. was hampered with decreasing C/N ratio. The inhibition effect might also have occurred from a combination of both these causes.

Both AD approaches in this work showed initial difficulties in coping with the new substrate type and the feed load. A successive drop of pH was observed after the beginning of the experiment starting from pH > 7.25 and reaching pH 6.9. In both cases, the values recovered and steadied in spite of maintaining the same operating conditions. It seemed that microorganisms in the digester sludge found a way to tackle the problem of coordinated degradation of RM after an adjustment period. Visual and olfactory analysis of digester sludge showed a viscous, black-colored slurry, emitting a strong and distinct sulfurous odor. A possible inhibition of microbial growth through increased salt concentration in the bioreactor, as suggested by Allen *et al.* (2013) [19] and Nielsen and Heiske (2008) [57], was ruled out since the values for conductivity were located within standard range [58]. However, thermophilic and mesophilic approaches showed differences regarding the trend of the conductivity curves, with a drop for the thermophilic and an increase for the mesophilic experiments.

Compared to the CH_4_ yields of maize silage or animal manure in the literature, the recovery rates of RM were only 20% (mesophilic) and 22% (thermophilic) in the case of maize silage and 24% (mesophilic) and 26% (thermophilic) in the case of animal manure [45], and were therefore much lower than expected for standard biogas substrates. The methane production was poorer than for the references but feasible with RM alone. Generally, AD of RM at mesophilic and thermophilic process temperature exhibited stability for the measured process parameters after an initial period of adjustment. However, a significant gain in methane recovery could not be observed using either mode of operation, a fact which matched the findings from Hansson (1983) [59] in AD of green macroalgae from southern Sweden.

### 3.6. Analysis of Fermentation Residue

The potential interest for further use of fermentation residue is always important to consider since it can present either a disturbing waste material or a valuable biofertilizer. RM fermentation residue showed, in general, uncritical concentrations of heavy metals. Only for cadmium were elevated concentrations measured. Elevated cadmium concentrations were also detected in the digestate from AD of Baltic Sea macroalgae originating from Trelleborg in Sweden, which was designed for biofertilization [60]. The vicinity of the collection sites indicates an elevated degree of pollution of the marine area location in regards to cadmium concentration. Methanogenic microorganisms are generally sensitive to heavy metals [46], but the intensity can differ according to the microbial pool composition and the presence of metal-tolerant or metal-resistant species [28]. Macroalgae and seaweed can act as an absorption structure for heavy metals through mitigation of environmental concentration by increased uptake in the algal biomass [40,61]. This is particularly the case in industrialized coastal areas, for instance, the enclosed Baltic Sea, which exhibits high heavy metal concentrations and low distribution through tidal activity [62]. However, looking from a different angle, the intensified eutrophication or hypertrophication presents an advantage for bioremediation due to accelerated heavy metal fixation through accelerated algae growth. Using RMR as fertilizer is generally possible but its suitability depends on the nutritional needs of the soil. RMR is rich in nitrogen and sulfur, two of the essential nutrients important for fertilizer application. A problem might lie in the increased concentration of heavy metals, particularly cadmium. Filipkowska *et al.* (2008) [62] noted that beached macroalgae from the Sopot area are not recommended for use as agricultural biofertilizer due to enhanced stress from contaminants. However, there are attempts to develop new strategies for heavy metal detoxification of fermentation residues to allow their further usage in agriculture [60,63].

## 4. Experimental Section

### 4.1. Macroalgae Biomass Rügen-Mix and Maize Silage

Macroalgae were harvested from the beach area in Juliusruh (54° N, 13° E) on the island of Rügen, Germany, in August 2011. Within one day, the humid biomass was transported to Bremen and dried at 53 °C for conservation purposes. The biomass was cleared from most of its coarse debris, such as stones, shells, and other objects. Optical analysis allowed the distinction of three different algal groups, namely red algae (~80%–90%, probably from the *Spermothamnion* family), green algae (~5%–15%, probably from the *Chaetophorales* family), and eelgrass (~2%–5%, probably *Zostera marina*). However, the biomass mixture was strongly agglutinated and exact analysis of species involved and a calculation of the exact share was difficult. The biomass consistency showed a tightly wedged and carpet-like structure where single algae could not be separated from each other. An elementary analysis was done to check the composition of the algal biomass. The amount of VS in the dried biomass was 68.6% and water content was 75%. For simplified technical application in the bioreactor, dried macroalgae were crushed and homogenized in a grinding mill (Grindomix 200, Retsch, Haan, Germany) and sieved to a particle size of <0.5 mm before use. The name Rügen-Mix was chosen due to the origin of location of the biomass.

Maize silage for the laboratory experiments was obtained from a local biogas plant operator in Osterholz-Scharmbeck/Lower-Saxony, Germany. The maize silage consisted of moistly and coarsely shredded whole-plant particles (approximately 0.5–2 cm particle size). After sampling, the biomass was divided into smaller shares and stored at −20 °C until further use.

### 4.2. Inoculum Sludge

The inoculum for the batch experiments was obtained by mixing seeding sludge from a waste water treatment plant in Farge/Bremen, Germany, and a commercial biogas plant in Osterholz-Scharmbeck/Lower-Saxony, Germany. These plants were running on maize silage and cattle manure and operated at temperature conditions of 35 °C and 40 °C, respectively. The fresh inoculum was mixed with a small quantity of fermenter sludge adapted to macroalgae substrate used in previous batch experiments at a ratio of 3:1. Coarse material in the seeding sludge, such as fibers, impairing equipment handling during the experiments, was removed prior to use. To reduce the endogenous methane potential of the inoculum, the bioreactors were anaerobically incubated for one week prior to the addition of substrate.

The inoculum for the continuous anaerobic digestion experiments originated from the same source as the one for the batch experiments. More attention was paid on carefully removing fibers and coarse material from the seeding sludge to avoid interference with the agitator in the bioreactor and further technical problems with the equipment.

### 4.3. Flue Gas Condensate

The FGC was obtained from a middle caloric power plant in Bremen, Germany (swb AG), which uses paper, wood, synthetics, and residual package material as combustibles. The clear FGC showed a waterish consistency and had a pH 0.92. Based on the information of the source material (exhaust gases from combustion), FGC is supposed to mainly contain inorganic acids such as sulfuric/sulfurous acid and nitric/nitrous acid. An exact analysis of the FGC was not performed. The FGC was stored at room temperature until further use.

### 4.4. Acid Hydrolysis Pretreatment

Macroalgae biomass was generally pretreated by mechanical shredding before further use. After this treatment, the sample biomass consistency was dry and powdery. For acid hydrolysis, the powdery material was immersed into the acidic solution and the heterogenic suspension was heated and thoroughly mixed using a thermo-controlled laboratory shaker for stirring (HT Aquatron, Infors AG, Bottmingen, Switzerland, 175 rpm). After cooling, the pH of the solution was adjusted with NaOH to a value between 6.8 and 7.1 if necessary. The prepared substrate suspensions were stored at 4 °C until use in the experiments. The entire macroalgae-liquid suspension was poured into the digester during the feeding procedure to guarantee addition of all organic matter, solid and dissolved, in the supernatant. An overview of the different types of pretreatment applied in this work is displayed in Table 6.

**Table 6:** List of pretreatment conditions applied in this work for acid hydrolysis of Rügen-Mix. Name of the sample (Name), PT reaction time (Reaction time), PT temperature (Temperature), PT medium (Medium), and concentration of the medium (Concentration).

Name	Reaction time	Temperature	Medium	Concentration
RM-U; U	-	-	H_2_O	-
0.05 M	2 h	80 °C	HCl	0.05 M
0.1 M	2 h	80 °C	HCl	0.1 M
0.2 M	2 h	80 °C	HCl	0.2 M
30 min	30 min	80 °C	HCl	0.2 M
60 min	60 min	80 °C	HCl	0.2 M
90 min	90 min	80 °C	HCl	0.2 M
HCl	2 h	80 °C	HCl	pH 1.2
FGC	2 h	80 °C	FGC	pH 1.2
H_2_O	2 h	80 °C	H_2_O	-
100	2 h	100 °C	HCl	0.2 M

### 4.5. Biomethane Potential Tests and Batch Array

The batch arrays consisted of four types of subunits in successive order: biodigester, CO_2_ scrubbing unit, gas drying unit, and gas volume sensor. NaOH (3 M) was used to remove CO_2_ and H_2_S from the biogas, leaving only biomethane for gas volume measurement. Thymolphthalein indicator (0.4%) was added to NaOH to monitor the efficiency of CO_2_ absorption performance. The volumetric CH_4_ recordings were conducted with a gasUino apparatus and corrected to standard conditions (0 °C and 1013.15 hPa). The gas counter functions on a liquid displacement principle where 75% NaCl solution (pH 1) was used to prevent losses through gas diffusion. Butyl rubber tubing was used for the connections to avoid gas leakage [20].

The BMP tests were conducted at a mesophilic temperature (37 °C ± 1 °C). All experiments were performed in triplicate (2000 mL bottles), including one positive (microcrystalline cellulose (mcc)) and one negative (inoculum alone) control per experiment. Digester bottles were filled with 1600 mL of inoculum seeding sludge for pre-incubation. Feeding was performed with addition of untreated or pretreated 400 mL substrate-media suspension (neutralized with NaOH to pH 6.5–7.0 when needed) to a total volume of 2000 mL. Following the VDI 4630 guideline, the final VS value of the inoculum sludge was chosen between 1.5% and 2%. Accordingly, the amount of substrate added was set to VS (substrate) = 0.5 × VS (inoculum) [44]. Digester tanks were flushed with N_2_ for 2 min after feeding to create initial anaerobic conditions. Methane production was recorded for at least 22 days. It was required that positive control (mcc) achieved at least 75%–80% of its maximum value (373 mL·g^−1^ VS, aligned to indications in VDI 4630 protocol and LfU guidelines) for correct validation of the acquired data set [43,44].

### 4.6. Setup Continuous Stirred Tank Reactor (CSTR) 2 L

A 2 L Biostat B fermenter (B. Braun, Melsungen AG, Melsungen, Germany) was used to perform continuous AD experiments. The setup consisted of a stirred digester tank with permanent agitation and a heating jacket to keep steady process temperature (mesophilic at 37 °C ± 1 °C and thermophilic at 53 °C ± 1 °C). The gas outlet was connected via butyl rubber tubes to a subsequent CO_2_ scrubbing unit, a gas drying unit and finally to a gasUino gas volume sensor (identical order as in the batch arrays). A process electrode for measurement of pH was permanently inserted in the digester tank. An in- and outlet pipe was used for sludge sampling and substrate feeding. Sampling and feeding were manually performed once per day (every 24 h ± 3 h) using a technique which avoided sludge spilling or loss of biogas and head space pressure.

### 4.7. Analytical Methods and Calculations

#### 4.7.1. Measurement of Volatile Solids and Total Solids

Total solids (TS) and VS values were determined by drying and ashing samples at 105 °C and 550 °C, respectively, for 24 h (P300, Nabertherm, Lilienthal, Germany). The data points were the average of three individual values (*n* = 3). Error bars and deviation (±) were calculated from the standard deviation of triplicate values.

#### 4.7.2. Data Treatment from Methane Production

The total methane volume and the cumulative methane production in batch experiments were calculated by subtracting the average blank sample values accordingly. The methane production was recorded over time and volume detection was adjusted to standard conditions using the normalization formula used by Barbot *et al.* (2014) [20]. For batch experiments, all given data points consist of an average of three individual values (triplicates, *n* = 3). Error bars and deviation (±) were calculated with the standard deviation of triplicate values.

#### 4.7.3. Calculations for Comparison of Methane Formation Dynamics in Batch

A combination of first and second order kinetics was used to investigate the dynamics of methane formation for the respective approaches. Equation (1) was used to determine the decay constants *k* and to calculate the respective points in time (days) when 50%, 70%, and 90% of the total BMP were reached. The modified Gompertz Formula (2) was used to determine the lag phase Δ for each experimental approach [19].

(1) $$G\left(t\right)=G_{0}\;\times (1-e^{-kt})$$

(2) $$G\left(t\right)=G_{0}\;\times [-e\left[\left(\frac{u}{G_{0}}\right)\times \left(\Delta -t\right)+1\right]]$$

where *G(t)* is the cumulative CH_4_ yield at time *t* (mL CH_4_·g^−1^ VS), *G_0_* is the total BMP of the substrate (mL CH_4_·g^−1^ VS), *k* is the CH_4_ production decay rate constant (days^−1^), *t* is the time of BMP test in days and Δ is the lag phase (days).

## 5. Conclusions

The beach macroalgae biomass Rügen-Mix represents a possible substrate for use in biogas plants. However, the biomethane recovery rate was shown to be considerably inferior when compared to popular feedstock substrates, such as maize silage or cattle manure. RM can be subject to acid hydrolysis pretreatment which successfully increased the methane conversion efficiency and the methane formation rate. The acid hydrolysis pretreatment could be conducted with industry-grade acid HCl but was equally successful and functional with the use of the liquid acidic waste product FGC, which is consequently disposed of in this process step. The long-term AD of single RM in mesophilic and thermophilic process operation was successful for three hydraulic retention times, but methane recovery was lower than that of the batch trials. The main reason for insufficient performance was likely to be the critical sulfur concentrations in the RM substrate, in the bioreactor, and, consequently, in the RM digestate. It is recommended to use RM in co-digestion with trace-element-deficient and carbon-rich feedstock biomass to dilute the high sulfur concentration and to complement the facing deficiencies. Small-scale and mobilized container-shaped plug-flow bioreactors (dry fermentation) used in agricultural biogas plants might present a suitable technique to manage the high content of total solids and coarse biomaterial of RM.

The co-digestion of terrestrial energy crops with 25% seaweed share (by weight) similar to pretreated RM could decrease land-use for bioenergy in Germany by approximately 290,000 hectares for energy crops and approximately 211,000 hectares for maize. This represents 10%–14% of cultivated land where renewable resources are generated for energy use [64].
